# In Vitro Probiotic Potential of Lactic Acid Bacteria Isolated from Brazilian Dry-Cured Loin (Socol)

**DOI:** 10.3390/microorganisms13122749

**Published:** 2025-12-03

**Authors:** Felipe Coser Chow, Gustavo Lucas Costa Valente, Viviana Patrícia Fraga Santos, Carla Ferreira Soares, Henrique César Pereira Figueiredo, Silvana de Vasconcelos Cançado, Tadeu Chaves Figueiredo, Marcelo Resende Souza

**Affiliations:** Escola de Veterinária, Universidade Federal de Minas Gerais (UFMG), Avenida Antônio Carlos, 6627, Belo Horizonte 30123-970, Brazilcarla.soares.vet@gmail.com (C.F.S.); silvanacancado@gmail.com (S.d.V.C.)

**Keywords:** Socol, MALDI-TOF/MS, bacterial antagonism, antimicrobial resistance

## Abstract

Socol is an artisanal meat product typical of Southeast Brazil. It is made from pork loin and ripened at room temperature. This work aimed to isolate, quantify, and identify lactic acid bacteria (LAB) in Brazilian dry-cured loin (Socol) as well as evaluate their in vitro probiotic potential. LAB were found in high amounts, varying from 2.5 × 10^3^ to 9.2 × 10^6^ CFU g^−1^. Eleven isolated bacteria were identified by Matrix-Assisted Laser Desorption Ionization–Time-Of-Flight/Mass Spectrometry (MALDI-TOF/MS). Of these, six strains (*Latilactobacillus brevis* SFC1A, *Latilactobacillus sakei* SFC2A, *Latilactobacillus curvatus* SFC6A, *Pediococcus acidilactici* SFC9A, *Latilactobacillus curvatus* SFC11A, and *Pediococcus pentosaceus* SFC11B) were submitted to in vitro probiotic tests. All were tolerant to bile salts and five of them to artificial gastric juice, and were all sensitive to tetracycline, chloramphenicol, and erythromycin. *L. brevis* SFC1A and *P. acidilactici* SFC9A inhibited all tested pathogenic bacteria and showed the broadest in vitro probiotic activity. Thus, they would be recommended as starter cultures for the elaboration of novel fermented meat products and to compose a bank of indigenous bacteria, as well as contribute to preserving Socol microbiota.

## 1. Introduction

Socol is a type of Brazilian dry-cured loin, which is an artisanal meat by-product that traces its origin to the Veneto region of Italy. Italian immigrants introduced its production in the 1890s in the municipality of Venda Nova do Imigrante (Espírito Santo, Brazil). In 2018, Socol was granted a Protected Designation of Origin [1]. Socol is unique, possessing a Geographical Identification that recognizes the aforementioned city as the only producer of this artisanal cured meat, guaranteeing its authenticity. This recognition values local culture, boosts the economy of the region, and adds value to the product. Commercial salt (NaCl, minimum 2.5%), garlic (*Allium sativum*), and black pepper (*Piper nigrum*) are used in Socol preparation. After seasoning, the loin is encased in porcine peritoneum or collagen-based casings. Fermentation occurs naturally, driven by microorganisms present in the raw material and the production environment, and the ripening process typically lasts for at least 45 days. Lactic acid bacteria (LAB) are essential contributors to the biochemical processes responsible for flavor formation and preservation in Socol [2].

LAB, as well as yeasts and molds, are found in fermented meat products [3]. They may help to standardize product properties and shorten curing times [4]. Some LAB are considered probiotics that inhibit the growth of undesirable microbiota, namely pathogenic and spoilage microorganisms [5,6]. According to the joint FAO/WHO report [7], probiotics are live microorganisms that, when consumed in adequate quantities, provide health benefits. Some LAB isolated from fermented meat products have been studied as novel starter cultures and probiotics [8]. Even though LAB may be present in Socol, they have been poorly studied, since few reports in the literature describe its microbial community and their beneficial roles [9].

Therefore, the objectives of this study were to isolate, enumerate, and identify LAB from Socol and evaluate their in vitro probiotic potential.

## 2. Materials and Methods

### 2.1. Sampling

Socol samples (n = 13) were purchased from the local market of Venda Nova do Imigrante, Espírito Santo State, Brazil. These products were produced in ten different facilities by members of the Association of Socol Producers (Assocol), which comprises twnty-five members, including ten family businesses and six agro-industries [10]. The ripening process was conducted at room temperatures ranging from 16 °C to 26 °C.

### 2.2. Isolation and Enumeration of Lactic Acid Bacteria (LAB)

Twenty-five grams of Socol were collected, including interior and exterior parts. Then, 250 mL of phosphate-buffered saline (PBS) were added to prepare the 10^−1^ dilution. Subsequently, decimal dilutions were made using the same diluent. An aliquot of 100 µL was spread-plated on MRS agar (Merck, Darmstadt, Germany), which was incubated under aerobioses at 37 °C for 48 h [11] for LAB isolation and enumeration. Colonies with different morphotypes were characterized and identified by Matrix-Assisted Laser Desorption Ionization–Time-Of-Flight/Mass Spectrometry (MALDI-TOF/MS), using a Bruker Microflex MALDI Biotyper 2.0 (Bruker Daltonics, Billerica, MA, USA), as previously described by Assis et al. [12]. The criterion recommended by the manufacturer was as follows: a score ≥2000 indicates identification at the species level, <2000 and ≥1700 at the genus level, and <1700 is not reliable [13].

### 2.3. In Vitro Assessment of the Probiotic Potential of LAB

All analyses were performed in duplicate with three replications.

#### 2.3.1. Tolerance to Artificial Gastric Juice

The in vitro tolerance to artificial gastric juice was assessed following the procedures described by Neumann [14] and Silva et al. [15]. Lactobacilli samples were cultured in MRS broth (Oxoid, Basingstoke, Hampshire, UK) for 24 h at 37 °C under aerobic conditions. After two activations, the samples were centrifuged at 10,000× *g* for one min to form the pellet. Then, the bacterial sediment was dissolved in ultra-pure water, and 1 mL of each was transferred to microtubes. They were diluted 10× in artificial gastric juice (pepsin 3 g L^−1^, pH 2.0) and 10× in control (0.9% saline, pH 7.0). Afterward, 200 µL of the culture bacteria were transferred to the wells of a 96-well microplate and incubated in a spectrophotometer (Microplate Spectrophotometer System 47 SpectraMax 340—Molecular Devices, Sunnyvale, CA, USA) for 12 h at 37 °C. OD620 nm was measured every 30 min to determine the absorbance. The percentage of growth inhibition was calculated in GraphPad Prism 5.0 using the formula (1 − SG/CT) × 100, in which SG and CT correspond to the areas under the growth curve of bacteria treated with artificial gastric juice and the control, respectively. According to the criteria proposed by Acurcio et al. [16], results were classified as follows: tolerant (<40%), moderately tolerant (40–80%), and sensitive (>80%).

#### 2.3.2. Tolerance to Bile Salts

The tolerance to bile salts was assessed following Walker and Gilliland [17] and Silva et al. [15]. Lactobacilli cultures were grown in MRS broth (Difco, Difco, Detroit, MI, USA) for 24 h at 37 °C under aerobic conditions. Subsequently, 1 mL of each culture was transferred to microtubes and diluted to 4% (*v*/*v*) in MRS broth (Difco). Immediately afterward, 100 µL of the diluted culture were transferred to a well of a 96-well microplate containing 100 µL of MRS broth (Difco), and another 100 µL were transferred to a second well containing 100 µL of MRS broth (Difco) supplemented with 0.3% (*w*/*v*) bile salts (Oxgall^®^, Difco). The microplates were incubated in a spectrophotometer (SpectraMax 340 System; Molecular Devices, Sunnyvale, CA, USA) for 12 h at 37 °C. Absorbance was recorded as OD620 nm every 30 min. The calculation of growth inhibition and the interpretation of results were performed as described in the previous section.

#### 2.3.3. Spot-on-the-Lawn Antagonism

The antagonistic activity of the LAB against the indicator bacteria was evaluated using the spot-on-the-lawn assay described by Tagg et al. [18]. The isolates were cultured in MRS broth (Difco) for 24 h at 37 °C under aerobic conditions. Then, 5 µL of each culture was spotted onto MRS agar (Difco). After incubation at 37 °C for 48 h under aerobic conditions, the bacterial cells were inactivated by exposure to chloroform for 20 min, and the residual chloroform was allowed to evaporate. The Petri dishes were then overlaid with 3.5 mL of BHI or MRS semi-solid agar (0.7%) (Difco), previously inoculated with 10 µL of a 24 h culture of *Escherichia coli* ATCC 25922. *Listeria monocytogenes* ATCC 15313, *Salmonella enterica* var. Typhimurium ATCC 14028, and *Staphylococcus aureus* ATCC 33591. *Latilactobacillus curvatus* SFC11A isolated from Socol was also used as an indicator strain. We also used a chloroform blank control in order to confirm the results. After 24 h of incubation at 37 °C under aerobic conditions, the plates were examined to investigate and measure the inhibition halo. The choice of pathogenic bacteria was due to their association with foodborne diseases, while the SFC11A strain was a non-pathogenic microorganism. The values of the inhibition haloes were measured with the aid of a digital caliper (Mitutoyo Digimatic Caliper, Mitutoyo Sul Americana Ltd.a, Suzano, São Paulo, Brazil).

#### 2.3.4. Antimicrobial Resistance Profile

The antimicrobial resistance evaluation was performed using the disk diffusion methodology based on Charteris et al. [19]. The LAB colonies previously grown on MRS agar (Oxoid) under aerobiosis for 24–48 h at 37 °C were transferred to transparent polystyrene tubes containing 3.5 mL of 0.85% buffered saline. The bacterial suspension was adjusted to a turbidity corresponding to 0.5 on the McFarland scale (1.5 × 10^8^ CFU mL^−1^). Then, swabs from this solution were inoculated onto the surface of MRS agar (Oxoid). Soon after, the antimicrobial disks were placed on the agar and the plates were incubated under aerobiosis at 37 °C for 24–48 h. Antimicrobial disks (Laborclin, Pinhais, Paraná, Brazil) were as follows: ceftazidime (30 μg), clindamycin (30 μg), ciprofloxacin (5 μg), erythromycin (15 μg), gentamicin (10 μg), oxacillin (1 μg), penicillin G (10 UI), tetracycline (30 μg), vancomycin (30 μg), chloramphenicol (30 μg), amikacin (30 μg), and sulfazotrim (25 μg). Quality control of disks was performed using *E. coli* ATCC 25922. The diameters of the inhibition halos were measured using a pachymeter, including the diameter of the disk, and were classified as resistant, moderately sensitive, and sensitive [19].

## 3. Results and Discussion

### 3.1. Enumeration and Identification of Lactic Acid Bacteria (LAB)

The enumeration of LAB varied from 2.5 × 10^3^ to 9.2 × 10^6^ CFU g^−1^ in Socol samples. LAB are naturally present in meat or natural casings and ferment the sugars present in the medium into lactic acid. They also decompose molecules of protein, fat, and carbohydrates in the meat to generate aromatic substances such as alcohols, aldehydes, acids, and esters, as well as amino acids and small-molecule peptides [20]. Fermentation products determine sensory characteristics such as flavor, texture, and color that contribute to the identification of the artisanal product. Furthermore, the acidification (pH ~5) of the medium helps preserve the food. LAB must be present at values of at least 10^5^ CFU g^−1^ to compete, multiply, and promote characteristic acidification [21,22]. Few scientific papers describe LAB enumeration in Socol. Mutz et al. [2] reported counts of 7.9 × 10^4^ CFU g^−1^. Furthermore, the LAB present in lower counts (<10^5^ CFU/g) probably could not play fermentation roles, since the amount found in some Socol samples was below the functional threshold [8]

LAB species isolated from Socol samples were as follows: *Latilactobacillus brevis* SFC1A, *Latilactobacillus sakei* SFC2A, *Enterococcus faecalis* SFC4A, *Latilactobacillus curvatus* SFC6A, *Enterococcus faecalis* SFC7A, *Pediococcus acidilactici* SFC9A, *Enterococcus faecalis* SFC10A, *Enterococcus faecalis* SFC10B, *Latilactobacillus curvatus* SFC11A, *Pediococcus pentosaceus* SFC11B, and *Enterococcus faecalis* SFC13A. In the literature consulted, no description of LAB species present in Socol was found. *L. brevis*, *L. curvatus,* and *L. sakei* were also reported in the following artisanal fermented meat products: Nem Chua in Vietnam [23]; artisanal salami Ciauscolo in Italy [24]; dehydrated sausage in Argentina [25]; and Pancetta and Prosciutto in Ireland [26]. *Pediococcus* and *Enterococcus* were also described in Nem Chua and Ciauscolo.

### 3.2. Evaluation of the In Vitro Probiotic Potential of Microorganisms Isolated from Socol

#### 3.2.1. Tolerance to Gastric Juice and Bile Salts

The tested LAB were tolerant to the adverse conditions of the media used, demonstrating in vitro probiotic potential (Table 1).

The tolerance to acid conditions of the studied LAB may also be explained by their origin, since they were isolated from Socol, which is an acidic food. The low pH present in this meat product favors acid-tolerant bacteria to survive and dominate microbiota. Despite some variation in the results, none of the analyzed bacteria were classified as sensitive to bile salts, which is a desirable characteristic for the screening of probiotic microorganisms. It is important to note that the action of bile salts contributes to the removal of pathogenic bacteria from the intestinal environment, as they act as detergents on the microbial plasmatic membrane and can also cause damage to bacterial DNA. However, these phenomena are not selective and affect desirable bacteria. The tolerance of LAB to bile salts is associated with the production of the enzyme bile salts hydrolase (BSH), which deconjugates bile salts and reduces their detergent action [27,28].

Federici et al. [24] and Kong et al. [29] also described the tolerance to gastric juice and bile salts of *Lactobacillus* and *Pediococcus* isolated from Italian Ciauscolo and Harbin Dry Sausage from Northwestern China, respectively. Moreno et al. [30] reported that *Latilactobacillus plantarum* CTC 368 and CTC 469, isolated from meat (raw, cooked, and cured) and meat products (raw, cured, and dehydrated), were tolerant to the challenge of acidic medium (pH = 2), and the second strain was tolerant to bile salts (0.1%).

#### 3.2.2. Bacterial Antagonism (Spot-on-the-Lawn)

*L. brevis* SFC1A and *P. acidilactici* SFC9A were able to inhibit the growth of all indicator bacteria (Table 2). LAB may have inhibited pathogens by reducing the pH of the medium and producing bacteriocins. Four of the six tested LAB inhibited the growth of the indicator LAB isolated from Socol, compromising their use as starter cultures to be included in fermented products. Lucumi-Banguero et al. [31] reported the inhibition of pathogens by lactic acid produced by LAB in chorizo in Colombia, while Kaveh et al. [32] described the biopreservation of meat and meat products fermented by LAB and their metabolites.

The inhibition of *L. curvatus* (SFC11A) probably occurred by the production of bacteriocins, as LAB were isolated from the fermented meat product, which has an acidic pH. Rzepkowska et al. [33] tested different LAB isolated from Polish fermented meat products and identified the ability of *L. brevis* SCH5 and SCH6 to produce bacteriocins and similar substances. Laranjo et al. [34] mentioned that pediocin has an inhibitory effect against *L. monocytogenes*, as observed in the present study in which *P. acidilactici* SFC9A and *P. pentosaceus* SFC11B inhibited the pathogen.

*S. aureus* ATCC 33591 was only inhibited by *L. brevis* SFC1 and *P. acidilactici* SFC9. Parlindungan et al. [26] isolated and identified LAB in Pancetta and Prosciutto and found antagonism of those bacteria against *S. aureus* (NCDO949) and other pathogens, such as *Klebsiella aerogenes* (NCIMB10102), *Pseudomonas aeruginosa* (PA01), and *Listeria innocua* (UCC3).

#### 3.2.3. Antimicrobial Resistance Profile

In general, the tested LAB showed promising results from a probiotic point of view in the antibiogram, as most of them were sensitive to the used drugs (Table 3). *L. sakei* SFC2A demonstrated the best results since it was resistant to only four of the twelve pharmacological bases tested.

Five of the six tested LAB were resistant to vancomycin. In general, *Lactobacillus* spp. and *Pediococcus* spp. show intrinsic resistance to vancomycin due to the replacement of the terminal D-alanine residue by D-lactate or D-serine in muramylpentapeptide [6]. *L. sakei* SFC2A was sensitive to that antimicrobial, as also reported by Toomey et al. [35] in strains of *Limosilactobacillus reuteri, Lacticaseibacillus paracasei,* and *Latilactobacillus curvatus.* The vancomycin resistance genes of *Lactobacillus* spp. are chromosomally located and not easily transferable to other bacterial genera [36].

Results of multidrug resistance to antimicrobials were observed in some LAB isolated from Socol, revealing a public health problem. However, over the years, the resistance of bacteria to antimicrobials has been reported by several authors when analyzing LAB isolated from meat products [6,35,37].

Based on the evaluation of in vitro tests, *L. brevis* SFC1A and *P. acidilactici* SFC9A demonstrated probiotic activity, since they showed tolerance to bile salts and artificial gastric juice; inhibited *E. coli* ATCC 25723, *L. monocytogenes* ATCC 15313, *Salmonella enterica* var. Typhimurium ATCC 14028, and *S. aureus* ATCC 33591; and were sensitive to most of the antimicrobials used.

## 4. Conclusions

Brazilian dry-cured loin (Socol) presents microbiota including potentially probiotic microorganisms such as strains of LAB that can contribute as starter and flavor-producing cultures. Among the isolated LAB, *L. brevis* SFC1A and *P. acidilactici* SFC9A showed in vitro potential probiotic results, demonstrating their potential to antagonize undesirable bacteria in Socol. The isolated LAB are being kept, composing a bank of indigenous cultures which will be tested in the future as starter cultures for fermented meat products, as well as to preserve the native microbiota of Socol.

## Figures and Tables

**Table 1:** Means of inhibition percentage and classification according to tolerance to artificial gastric juice (pH 2.0) and bile salts (0.3% oxgall) of LAB isolated from Brazilian dry-cured loin (Socol).

Lactic Acid Bacteria	Gastric Juice	Bile Salts
*L. brevis* SFC1A	22.53 ± 12.86 (T)	−24.19 ± 3.87 (T)
*L. sakei* SFC2A	50.55 ± 14.05 (MT)	31.69 ± 16.81 (T)
*L. curvatus* SFC6A	1.52 ± 0.95 (T)	−15.03 ± 10.50 (T)
*P. acidilactici* SFC9A	−19.42 ± 16.52 (T)	−18.39 ± 8.09 (T)
*L. curvatus* SFC11A	−16.27 ± 2.95 (T)	−15.13 ± 10.59 (T)
*P. pentosaceus* SFC11B	15.23 ± 7.49 (T)	−8.82 ± 1.44 (T)

Analyses were performed in duplicate with three replications. T: tolerant (<40% inhibition); MT: moderately tolerant (40–80% inhibition), according to criteria established by Acurcio et al. [16].

**Table 2:** Means of inhibition haloes (mm) of lactobacilli isolated from Brazilian dry-cured loin (Socol) against indicator microorganisms by the spot-on-the-lawn assay.

Lactic Acid Bacteria	Means ± Standard Deviation of Inhibition Haloes (mm)				
	EC	LM	ST	SA	SFC11A
*L. brevis* SFC1A	44.72 ± 3.9	49.53 ± 6.7	32.58 ± 4.2	16.41 ± 14.4	32.35 ± 0.7
*L. sakei* SFC2A	41.67 ± 8.5	39.71 ± 1.4	7.77 ± 13.5	0	7.95 ± 13.8
*L. curvatus* SFC6A	31.69 ± 3.1	30.1 ± 4.8	0	0	0
*P. acidilactici* SFC9A	57.17 ± 4.2	62.57 ± 2.9	34.82 ± 2.5	32.25 ± 1.3	42.22 ± 5.9
*L. curvatus* SFC11A	30.35 ± 5.8	23.04 ± 4.7	0	0	0
*P. pentosaceus* SFC11B	50.37 ± 2.6	44.96 ± 6.6	17.65 ± 15.3	0	27.58 ± 5.3

Analyzes were performed in duplicate with three replications. EC (*Escherichia coli* ATCC 25723); LM (*Listeria monocytogenes* ATCC 15313); ST (*Salmonella enterica* var. Typhimurium ATCC 14028); SA (*Staphylococcus aureus* ATCC 33591); and SFC11A (*Latilactobacillus curvatus* isolated from Socol).

**Table 3:** Profile of antimicrobial resistance of lactobacilli isolated from Brazilian dry-cured loin (Socol).

Lactic Acid Bacteria	Antimicrobial											
	CEF	CLI	CIP	ERY	GEN	OXA	PEN	TET	VAN	CHL	AMI	SUL
*L. brevis* SFC1A	S	S	R	S	R	R	MS	S	R	S	R	R
*L. sakei* SFC2A	R	S	R	S	S	R	S	S	S	S	MS	R
*L. curvatus* SFC6A	S	MS	S	S	R	R	S	S	R	S	R	R
*P. acidilactici* SFC9A	S	S	R	S	S	R	S	S	R	S	R	R
*L. curvatus* SFC11A	S	S	MS	S	R	R	S	S	R	S	R	R
*P. pentosaceus* SFC11B	S	S	R	S	S	R	MS	S	R	S	R	R

Analyzes performed in duplicate with three replications. CEF: ceftadizyme (30 μg), CLI: clindamycin (30 μg), CIP: ciprofloxacin (5 μg), ERY: erythromycin (15 μg), GEN: gentamicin (10 μg), OXA: oxacillin (1 μg), PEN: penicillin G (10 UI), TET: tetracycline (30 μg), VAN: vancomycin (30 μg), CHL: chloramphenicol (30 μg), AMI: amikacin (30 μg), and SUL: sulfazotrim (25 μg). R: resistant; MS: moderately sensitive; and S: sensitive, according to Charteris et al. [19].

## Data Availability

The original contributions presented in this study are included in the article. Further inquiries can be directed to the corresponding authors.
